# Rapid mapping of visual receptive fields by filtered back projection: application to multi-neuronal electrophysiology and imaging

**DOI:** 10.1113/jphysiol.2014.276642

**Published:** 2014-09-29

**Authors:** Jamie Johnston, Huayu Ding, Sofie H Seibel, Federico Esposti, Leon Lagnado

**Affiliations:** 1 MRC Laboratory of Molecular Biology, Francis Crick AvenueCambridge, CB2 0QH, UK; 2 Sussex Neuroscience, School of Life Sciences, University of SussexBrighton, BN1 9QG, UK

## Abstract

Neurons in the visual system vary widely in the spatiotemporal properties of their receptive fields (RFs), and understanding these variations is key to elucidating how visual information is processed. We present a new approach for mapping RFs based on the filtered back projection (FBP), an algorithm used for tomographic reconstructions. To estimate RFs, a series of bars were flashed across the retina at pseudo-random positions and at a minimum of five orientations. We apply this method to retinal neurons and show that it can accurately recover the spatial RF and impulse response of ganglion cells recorded on a multi-electrode array. We also demonstrate its utility for *in vivo* imaging by mapping the RFs of an array of bipolar cell synapses expressing a genetically encoded Ca^2+^ indicator. We find that FBP offers several advantages over the commonly used spike-triggered average (STA): (i) ON and OFF components of a RF can be separated; (ii) the impulse response can be reconstructed at sample rates of 125 Hz, rather than the refresh rate of a monitor; (iii) FBP reveals the response properties of neurons that are not evident using STA, including those that display orientation selectivity, or fire at low mean spike rates; and (iv) the FBP method is fast, allowing the RFs of all the bipolar cell synaptic terminals in a field of view to be reconstructed in under 4 min. Use of the FBP will benefit investigations of the visual system that employ electrophysiology or optical reporters to measure activity across populations of neurons.

## Introduction

The visual system processes stimuli through a variety of spatial and temporal filters (Kuffler, 1973) and the results of this processing are described by the receptive field (RF) of neurons at different stages of the visual pathways. In the spatial domain, the RF is simply the area in visual space in which presentation of a stimulus alters that neuron's activity, while the temporal RF describes how the neuron responds over time to a given stimulus. Calculation of the spatio-temporal RF is a key step in understanding the computations that are carried out by the visual system (Berry *et al*. 1999; Baccus & Meister, 2002; Sher & Devries, 2012), and one of the fundamental aims of visual neuroscience is to understand how RFs with particular spatio-temporal properties are built by the neurons, synapses and dendrites of different neural circuits.

The first approaches to mapping RFs in the visual system employed spots or bars flashed at various locations while recording spikes from a single neuron (Hubel & Wiesel, 1959). This technique is time-consuming and cannot be applied systematically when recording activity from a population of neurons. A second approach is to calculate the spike-triggered average (STA) during presentation of a stimulus varying randomly in space and time across the retina, such as a flickering checkerboard providing a ‘white noise’ stimulus (Meister *et al*. 1994; Chichilnisky, 2001). The STA is then calculated for each neuron as the average stimulus preceding a spike, providing an estimate of the linear component of its RF. An important advantage of the STA is that the RFs of many neurons can be reconstructed in parallel, and this has been particularly useful in experiments monitoring activity across populations of retinal ganglion cells (RGCs) using multi-electrode arrays (MEAs) (Meister *et al*. 1994; Field *et al*. 2010; Marre *et al*. 2012; Sher & Devries, 2012). STA has been used to map RFs in neurons throughout the visual pathway, from the retina to the cortex (Hubel & Wiesel, 1962; DeAngelis *et al*. 1993; Meister *et al*. 1994; Martinez *et al*. 2005; McAdams & Reid, 2005; Lesica *et al*. 2007; Field *et al*. 2010; Sher & Devries, 2012; Zhao *et al*. 2013).

The use of the STA in visual neuroscience does, however, suffer from drawbacks:

- It can be time-consuming, especially if a cell fires at a low mean spike rate. An hour of stimulation with white noise may be insufficient to map some RFs (Marre *et al*. 2012).
- The temporal resolution with which the impulse response is measured is limited to the refresh rate of the monitor delivering the stimulus.
- White noise does not appear to be an effective stimulus for all ganglion cell types (Marre *et al*. 2012; Talebi & Baker, 2012). For example, a ganglion cell excited by spatially overlapping ON and OFF inputs will fire spikes when the stimulus is dark over its RF, but also when it is bright, and averaging these stimulus sequences will mask the RF.
- A growing number of experiments employ genetically encoded reporters to provide an optical readout of neural activity (Odermatt *et al*. 2012; Esposti *et al*. 2013; Yonehara *et al*. 2013; Nikolaev *et al*. 2013; Park *et al*. 2014) but performing a simple STA analysis on data acquired from such experiments is difficult because it requires accurate knowledge of either the kernel that equates spike rates with fluorescence changes (Smith & Häusser, 2010) or a weighting of the spike-triggered stimulus ensemble that takes account of the amplitude of the fluorescence change (Bonin *et al*. 2011).

Here we present an alternative approach to mapping RFs that addresses these drawbacks of the STA. The principle of the technique is the same as that used in medical tomography, where CAT scanners reconstruct 2-D images from 1-D line projections made by passing a series of parallel beams of high-energy radiation through the object (Brooks & Di Chiro, 1976). Each beam is used to measure the density of the part of the object it passes through, and the linear summations of the object's density along parallel beams at different angles is then used to reconstruct the whole object. The mathematical basis of this reconstruction is the Radon transform. Johann Radon showed that any function could be reconstructed exactly from an infinite set of such projections using the inverse of the Radon transform (Radon, 1917), and ‘filtered back projection’ (FBP) is a widely used algorithm for computing the inverse Radon transform using a finite number of beam angles (Brooks & Di Chiro, 1976). We have developed a similar approach to map the RFs of retinal ganglion cells (RGCs). In place of radiation beams, we used bars flashed onto the retina, and instead of assaying density we measured the strength of neuronal spike responses. We found that FBP overcomes several drawbacks of STA:

- It is fast. We show that a protocol of 4 min is sufficient to map RFs of many RGCs recorded on an MEA with a signal-to-noise ratio (SNR) similar to that achieved by 20 min of white noise stimulation followed by calculation of the STA.
- The impulse response can be calculated with a time resolution greater than 100 Hz because the temporal information is directly extracted from the electrophysiological data collected by the MEA rather than being limited to the refresh rate of the monitor delivering the stimulus.
- A number of subtypes of ganglion cell that are not effectively characterized by STA can be identified by FBP. These include neurons with low mean firing rates and those that are selective for orientation.
- The FBP method is easily applicable to data acquired with imaging methods. Using *in vivo* two-photon imaging of a genetically encoded calcium indicator we map the RFs of individual synaptic terminals of bipolar cells delivering the visual signal to RGCs through all strata of the inner plexiform layer.

The FBP is likely to be useful in many areas of visual neuroscience where knowledge of a neuron's RF is required. The time-scale on which RFs can be reconstructed across neuronal populations makes the FBP especially useful for experiments using multiple electrodes or imaging of fluorescent reporters.

## Methods

### Tissue preparation and electrophysiology

All procedures were carried out according to the UK Animals (Scientific Procedures) Act 1986 and approved by the UK Home Office. Adult goldfish or zebrafish were dark adapted for more than 30 min and decapitated followed by pithing. Further procedures were carried out under dim red light. Eyes were enucleated and retinae placed in AMES medium (Sigma, St. Louis, MO, U.S.A.) containing 5% hyaluronidase for 2 min to digest the vitreous humour. Retinae were then rinsed free of hyaluronidase and placed in a Petri dish where ∼25 mm^2^ patches were cut with a razor blade. One patch of retina was then placed, ganglion cell layer down, on a perforated 60 channel multi-electrode array (60pMEA200/30iR-Ti, Multichannel systems, Reutlingen, Germany) mounted on an MEA1060 amplifier equipped with a perfusion ground plate (Multichannel systems). The retina was held down by applying gentle suction via the perfusion ground plate with AMES perfused through the top of the chamber at ∼5 ml min^−1^. Extracellular recordings were acquired at 20 kHz on a PC running Windows 7.

### Visual stimuli

Visual stimuli were generated on a separate PC using custom software written in Matlab (MathWorks, Natick, MA, U.S.A.) utilising Psychophysics Toolbox (Brainard, 1997). For ganglion cell recordings, stimuli were displayed on an 852 pixel × 600 pixel organic light emitting diode (OLED) micro display (100080-01 eMagin, Bellevue, WA, U.S.A.). The screen was focused onto the retina through a single aspheric doublet lens, resulting in pixels measuring 4 μm × 4 μm on the retina; the mean irradiance of the retina was 8.6 nW mm^−2^. For the *in vivo* zebrafish recordings an Optoma PK320 pico projector (Optoma, Watford, Hertfordshire, U.K.) was modified to disable the power supply to the green and blue LEDs; this prevented light bleed through to the recording photomultiplier tube. The mean irradiance of the screen was 12.68 nW mm^−2^. Visual stimulation was synchronised with electrophysiology and imaging by recording the times of screen refreshes during stimulus presentations using custom-written code and a U3 LabJack digital-to-analog converter (Labjack, Lakewood, CO, U.S.A.).

### Spike sorting

Spikes were detected and sorted from raw voltage traces by clustering using Wave_Clus (Quiroga *et al*. 2004), a semi-automated spike-sorting algorithm written in Matlab (MathWorks). Spikes were detected by threshold crossings of 4 times the noise estimate. To aid discrimination of spikes from different neurons, spike waveforms were decomposed using wavelet analysis and the 10 wavelet coefficients whose distribution showed the greatest deviation from normality were used for clustering. Once the clusters were chosen the remaining spikes were added to the clusters by template matching. A detailed description of this method can be found in Quiroga *et al*. (2004).

### Mapping receptive fields with white noise and STA

The principle behind this method is reverse correlation of the spikes to the white noise stimulus, and it has been described in detail (Chichilnisky, 2001). For the white noise stimulus, a 60,000-frame movie of a flickering checkerboard was created where each square was 10 pixels × 10 pixels on the screen. The value assigned to each square was either 256 or 0, in a random sequence independent of the other squares. This movie was presented to the retina at a refresh rate of 30 Hz and lasted just over half an hour. The STA was calculated over 300 ms, equivalent to a stack of 10 frames of the movie. The resulting image stack provides spatial information, such as the location at which a stimulus is able to elicit the largest response, as well as temporal information, such as the delay from the presentation of stimulus to the occurrence of a spike. The resolution of the spatial field depends on the size of blocks used in the stimulus, which was 40 μm in our experiments. The resolution of the temporal field depends on the time between presentations of frames, which was 33.3 ms. To identify the frame containing the spatial RF, we first processed all the averaged frames with a Gaussian filter and then took the frame with the maximum amplitude pixel as the representation for the spatial RF. The centre of the RF was defined as the location of the maximum amplitude pixel, to which a two-dimensional Gaussian was fitted.

### Implementation of the FBP algorithm

We implemented the filtered back projection in Igor Pro (Wavemetrics, Portland, OR, U.S.A.), and the annotated code is available at http://www.igorexchange.com/project/FBP. Briefly, the function takes a data wave corresponding to a Radon transform of the RF and returns an image of the RF. In general terms, for a RF *f*(*x*,*y*), the Radon transform 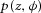 is:


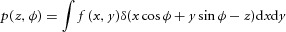


where δ is the Dirac delta function, *x* and *y* are the coordinates in Cartesian space, ϕ is the angle of projection and *z* is the distance perpendicular to ϕ. To reduce noise, the projections from each angle ϕ are filtered directly in the Fourier space to give 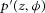. We used a Hamming filter set to 0.6. The filtered data were then used to reconstruct the RF, FBP(*x*,*y*), according to:


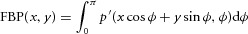


We implemented this using cubic spline interpolation to back project each angle onto the final image.

### Measuring SNR of spatial RFs

The SNR for spatial RFs was calculated as (signal – baseline)/noise. Signal was defined as the mean pixel value over the 3 × 3 pixel window containing the largest response, which corresponded to the centre of the RF. Noise was estimated by sliding a 10 × 10 pixel window over the RF and finding the position where the standard deviation was at a minimum. This area was always outside the centre of the RF. The baseline was the mean pixel value over this same 10 × 10 pixel window. Note that this method of calculating SNR was used for RFs reconstructed using both STA and FBP.

### Zebrafish

*Tg(–1.8ctbp2:SyGCaMP6)* fish were generated by co-injection of I-SceI meganuclease and endofree purified plasmid into wild-type zebrafish with a mixed genetic background (Thermes *et al*. 2002). The GCaMP6 variant (alternative name GCaMP3 variant 10.500) was kindly provided by L. Looger (Janelia Farm). This variant holds a T383S mutation in comparison to the commercially available GCaMP6-fast version (Addgene plasmid 40755) as described in Chen *et al*. (2013). For the ease of screening and to circumvent the low basal fluorescence of the reporter a myocardium-specific promoter (Huang *et al*. 2003) driving red fluorescent protein in the heart was co-cloned into the injection plasmid. Fish were raised and maintained under standard conditions as described (Nüsslein-Volhard & Dahm, 2002) on a 14 h light/10 h dark cycle. To aid imaging through the eye, fish used for two-photon imaging were heterozygous for the *roy orbison* mutation, which results in hypopigmentation (Ren *et al*. 2002) and treated with 1-phenyl-2-thiourea (200 μm final concentration; Sigma) from 1 day post fertilization (dpf) to reduce pigmentation further. All animal procedures were performed in accordance with UK Home Office guidelines and with the approval of the University of Sussex local ethical committee.

### Two-photon imaging

Fish were immobilised in 3% low melting point agar (Biogene, Kimbolton, Cambs, U.K.) with one eye pointing at a screen (see Fig.8*A*). Bipolar cell terminals were imaged *in vivo* using a Scientifica two-photon microscope (Scientifica, Uckfield, East Sussex, U.K.) equipped with a mode-locked Chameleon titanium–sapphire laser tuned to 915 nm (Coherent Inc., Sanat Clara, CA, U.S.A.) with an Olympus XLUMPLFLN 20× water immersion objective (NA 1, Olympus, Tokyo, Japan). Emitted fluorescence was captured through the objective. Scanning and image acquisition were controlled under ScanImage v.3.8 software (Pologruto *et al*. 2003).

**Figure 8:**
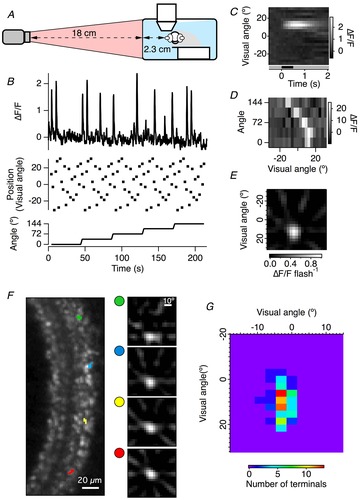
The FBP method is suitable for *in vivo* calcium imaging of zebrafish bipolar cell terminals *A*, diagram of the experimental setup. A zebrafish expressing SyGCaMP6(10.500) in bipolar cells was placed under the two-photon microscope objective, with one eye facing a screen positioned 2.3 cm away. Visual stimuli were presented using a rear projection system with the projector 18 cm away from the screen. *B*, the response of a single bipolar cell terminal to flashed bars at the positions and angles indicated. *C*, a peri-stimulus response diagram for a single bipolar terminal to −100% contrast bars flashed for 0.5 s onto different locations of the screen at a single angle. *D*, the integral of the response for each bar position and each bar angle, equivalent to the Radon transform. *E*, the RF of the bipolar terminal in *B* and *C* recovered using FBP. *F*, a field of view in the inner plexiform layer containing an array of bipolar synaptic terminals. Example RFs of 4 bipolar terminals are shown on the right. The locations of these terminals in the field of view are indicated by the colour code. Note that bipolar cell terminals situated at one side of the retina have RFs located at different locations from terminals situated at the opposite side. *G*, a map of the RF centre locations for all bipolar terminals detected in the field of view in *F*. The centre location was defined as the pixel in the RF with the maximum absolute value.

## Results

### Estimating visual receptive fields using the filtered back projection

To investigate how the FBP algorithm can be used to reconstruct RFs we began with a model RF represented by a 2-D Gaussian (Fig.1). A 1-D projection of the RF was generated by integrating each column of pixels from which the image was composed (Fig.1*A*). A series of such 1-D projections collected at evenly spaced angles corresponds to the Radon transform of the model RF (Fig.1*B*; Radon, 1917). Using this data set, the FBP algorithm was used to reconstruct the original RF (Fig.1*C*). In this test we used projections at five angles, which resulted in a streak artefact in the reconstructed image. Such artefacts are inherent to the FBP algorithm, and the most straightforward way to reduce them is to create projections at a larger number of angles (Brooks & Di Chiro, 1976). Nonetheless, this initial investigation indicated that an excellent reconstruction might be obtained using projections at just five equally spaced angles.

**Figure 1:**
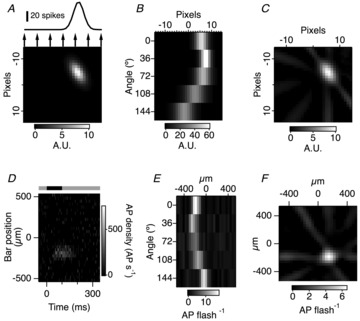
Principle of the filtered back projection *A*, a model RF, represented by a 2-D Gaussian, is shown in the 29 × 29 pixel image. A projection is obtained for a single angle by summing pixel values along parallel lines and plotting the sums against the positions of the lines. Note that the relative dimensions of the model correspond to the acquired data in *D–F*. *B*, projections from the model collected at five different angles. These data correspond to the Radon transform of the oval RF shown in *A*. *C*, the filtered back projection takes the Radon transform as input to reconstruct the original object. *D*, a peri-stimulus time histogram recorded from a ganglion cell in response to a −100% contrast bar flashed at different locations on the retina (average of 3 presentations, which took 240 s). The duration of the stimulus is indicated above, with grey for mean light and black for when the dark bar occurred. *E*, the Radon transform of the ganglion cell from *D* at five evenly spaced angles, the same as in *B*. *F*, the RF of the cell in *D* reconstructed using the filtered back projection; this RF was mapped in 240 s. A.U., arbitrary units.

Informed by the prediction of the model, we tested the utility of FBP in two common recording situations, multi-electrode arrays (Figs1–7) and multiphoton imaging of fluorescent reporter proteins (Fig.8). MEA recordings from RGCs were made in flat-mounts of goldfish retina. To acquire each 1-D projection we flashed bars onto the retina at different positions and plotted the number of evoked spikes *versus* location. Each bar was presented for 100 ms, at pseudo-random but parallel locations on the retina. We chose a bar width of 80 μm as this gave robust responses when flashed over ganglion cell RFs while also being narrower than the average RF of bipolar cells (Kaneko, 1973). The centres of bars flashed in adjacent locations on the retina were spaced 40 μm apart, giving a resolution of 40 μm to the final RF reconstruction, and the order of flashes was designed so that each presentation of the bar was not overlapping or adjacent to a preceding presentation to limit possible effects of local adaptation. The duty cycle for flashes was 500 ms.

**Figure 2:**
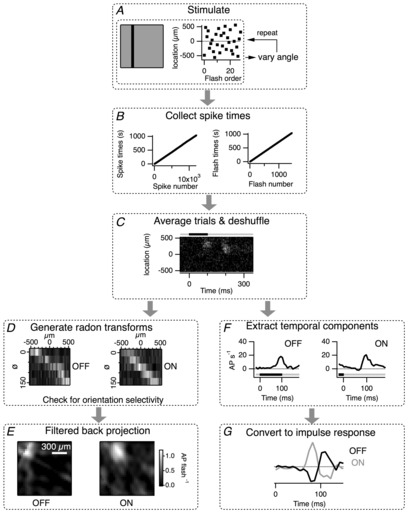
Workflow to reconstruct RFs using the FBP method *A*, a stimulus is generated using bars flashed onto the retina in a predetermined ‘random’ order. This is repeated at 5 or more evenly spaced angles. *B*, the spike times for each cell are extracted along with the onset of each flash. *C*, ‘GENERATE_PSTH’ creates an image stack in which each frame consists of the PSTHs for bars of a certain angle flashed at each location across the retina. Based on these PSTHs, the windows for parsing spikes are determined. For our data, 0–150 ms was used for examining the OFF component and 150–300 ms for examining the ON component; this takes into account the ∼50 ms delay inherent to phototransduction. *D*, ‘GENERATE_RADONS’ counts the number of spikes at each position and angle for the defined time windows of the OFF and ON components determined in *D*. The output is stored in an image plot corresponding to the Radon transform of the RF. *E*, ‘FBP’ reconstructs the spatial RFs for both the OFF and ON components. *F*, ‘TEMPORAL’ divides the PSTH into discrete time points and constructs a series of Radon transforms, which are then converted to a spatial temporal map of the RF from which the temporal response can be extracted (see Movie S1 for an example). *G*, the impulse response can be estimated by differentiating the temporal response in *F* (see Fig.5 for further details).

**Figure 3:**
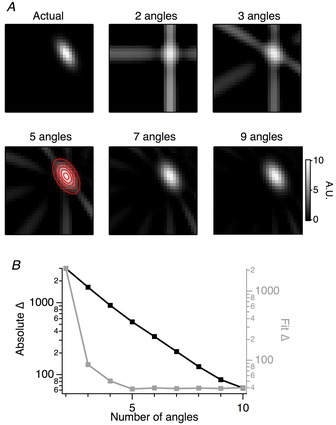
Five angles for the Radon transform are sufficient to reconstruct the RF *A*, Radon transforms, using different numbers of angles, were taken of the model RF (Actual). These transforms were then used with FBP to reconstruct the RFs shown (2–9 angles). With more angles, we achieve finer resolution and closer likeness to the original image. The red contours shown on 5 angles is a 2-D Gaussian fit to the reconstructed RF. *B*, comparison of the quality of reconstruction with different numbers of angles. Plotted in black are the absolute differences in pixel value between the reconstruction and the original model. Note that the difference between the reconstructed image and the original decrease with increasing angles. Plotted in grey is the absolute difference between a 2-D Gaussian fit to the reconstruction and the original model. Note that 5 angles are sufficient to capture the centre, size and shape of the object.

**Figure 4:**
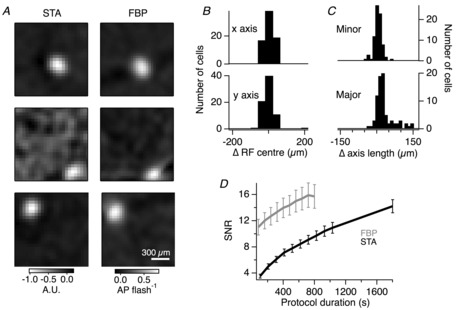
Comparison of spatial RFs reconstructed with FBP and STA *A*, three example cells were mapped with STA on the left and FBP on the right. FBP can map cells located on the edge of the detectable region just as well as cells located in the centre. *B*, histograms of the differences in *x* and *y* coordinates of the RF centres identified by FBP and STA. The histograms are centred on zero, showing that there is little difference between the centres mapped using either method. The centre coordinate measurements are constrained by the resolution of the image containing the RF. The physical size of each pixel in the reconstructed image is 40 μm × 40 μm. For example, when the STA-constructed RF has a centre coordinate on the image that is one pixel offset from the centre coordinate identified in the FBP-constructed field, the difference between them is 40 μm. *C*, histograms of the difference in length of the major and minor axes obtained from 2-D Gaussian fits to the RFs recovered from STA and FBP. The minor axis was centred on zero, but the major axis was slightly bigger when measured by FBP; the median difference on the major axis was 21 μm with an interquartile range of 7–58 μm. *D*, comparison of the SNR of the spatial RFs recovered with different stimulation durations for each method. Note for FBP only the number of averages was altered, the number of angles was always 5. The mean SNR and standard deviation is shown for the same 36 cells from a single retina.

**Figure 5:**
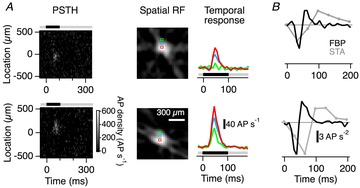
Temporal characteristics extracted from FBP *A*, examples of the procedure for two cells. Left: peri-stimulus time histogram (PSTH) at each bar position with the stimulus bar on top (average of 3 presentations, which took 240 s). Middle: reconstructed spatial RF. Right: the temporal response to the flashed bar for different locations within the RF. Colours correspond to regions shown in the spatial RF. *B*, the impulse response calculated by differentiation of the step responses in *A* shown in black. As a negative step was used, the polarity is reversed to give the impulse response. The impulse responses of the same cells calculated by STA are shown overlaid in grey.

**Figure 6:**
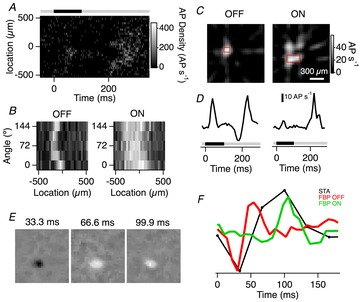
ON and OFF components can be separated by FBP *A*, an OFF component in response to the stimulus changing from mean light to a dark bar, as well as an ON component in response to the change back to mean light, can be observed in the PSTH of this cell (average of 3 presentations, lasting 240 s). *B*, Radon transforms for the OFF and ON components were constructed independently from windows at 0–150 ms and 150–300 ms, respectively. *C*, these can be used to reconstruct the OFF and ON spatial receptive fields independently; the ON component had a larger RF than the OFF component. *D*, the temporal responses for the OFF and ON RFs can be extracted separately, the temporal responses were extracted from the red boxes shown in *C*. *E*, 3 frames of the STA from the same cell showing 33.3 ms to 99.9 ms of the STA movie. *F*, comparison of the impulse response of the receptive field centre as measured by FBP and STA; note that the FBP extracts separate OFF and ON impulse responses and at a higher temporal resolution.

**Figure 7:**
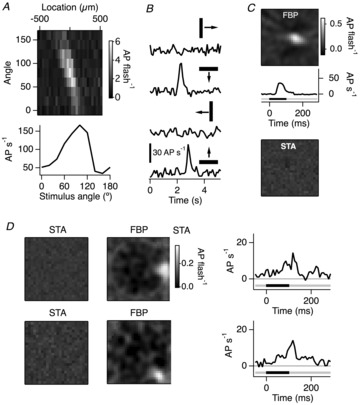
FBP recovers more RFs than STA *A*, the Radon transform over 9 angles is shown on top for an orientation-selective cell. The peak response *vs*. the angle of the bar is shown below. The response is strongest for angles around 90 deg. *B*, the same cell responding to a bar moving in 4 different directions. No response is seen for bars moving at either 0 or 180 deg, but clear responses are seen for bars moving along 90 or 270 deg. *C*, the RF of the cell recovered with the FBP and the STA methods. Note that the RF is not apparent in the STA image. The temporal component measured with FBP is shown below. *D*, two examples of cells with weak responses that were recovered by the FBP method but not by the STA method with their temporal responses shown to the right.

In the example shown in Fig.1*D* and *E* a sequence of 29 positions was presented 3 times for each angle, and data were collected over a period of 240 s. The peri-stimulus time histogram (PSTH) was then plotted for each bar position (Fig.1*D*), and the strength of the response quantified as the number of spikes over a 150 ms time window beginning at the start of the presentation, which can be seen to correspond to the integration time of the flash response. This single measure was used to generate each point in the line projection, and then this procedure was repeated for bars at other angles to produce each projection of the Radon transform. Projections at five equally spaced angles between 0 deg and 144 deg are shown in Fig.1*E*. They do not align because the RF is not in the centre of the visual space being sampled, and they are not of equal strength because the RF is asymmetric.

The final step in the procedure was to reconstruct the RF by calculating the filtered back projection from the collection of line projections, and the result is shown in Fig.1*F*. The quality of the reconstruction obtained in 240 s using five angles of projection appears similar to that from the model in Fig.1*C*. These results demonstrate that a simple bar stimulus can be used to acquire the Radon transform of a neuron's RF and that the FBP can then reconstruct the spatial detail of this RF.

A more detailed summary of the steps taken to obtain the spatial and temporal RFs using the FBP method is shown in Fig.2, which also includes examples of the data at each step. This analysis was carried out using Igor Pro 6 (Wavemetrics) and the functions in inverted commas are available at Igor Exchange (http://www.igorexchange.com/project/FBP).

A stimulus is generated using bars flashed onto the retina in a predetermined ‘random’ order; this is repeated at five or more evenly spaced angles (Fig.2*A*). The spike times for each cell are extracted along with the onset times of each flash (Fig.2*B*). Taking the known order of the flashes and the flash onset times, the spike time data are parsed out and deshuffled to generate a stack of peri-stimulus time histograms (PSTHs). Each frame in the image stack corresponds to the data from one angle and shows the PSTH elicited for each bar position. The script ‘GENERATE_PSTH’ automates this procedure (Fig.2*C*). From the PSTH, Radon transforms can be constructed by binning the spikes over a temporal window. To construct just the spatial RF we binned the spikes over the total response time, which for our data was 0–150 ms for the OFF component and 150–300 ms for the ON component. Spikes are counted in these windows and plotted against their location and angle to create the Radon transforms for both the OFF and ON components. Orientation selectivity can be assessed at this stage by examining the Radon transforms (see Fig.7). The script ‘GENERATE_RADONS’ automates this procedure (Fig.2*D*). The FBP algorithm takes the Radon transform and reconstructs the spatial RFs for both the OFF and ON components. We implemented the FBP algorithm with interpolation using cubic splines and a Hamming filter, routinely set to 0.6. The script ‘FBP’ performs the filtered back projection. (Fig.2*E*). From the PSTH the temporal components of the RF can also be extracted. Instead of binning spikes across the total response time, a ‘binning’ window (set to 8 ms) is passed across the PSTH creating a series of Radon transforms which are then converted by FBP to an image stack showing how the response to the stimuli varies in both space and time. The script ‘TEMPORAL’ automates this procedure (Fig.2*F*). Once the temporal components have been extracted, differentiation of the appropriate region will give an estimate of the impulse response (Fig.2*G*; see below).

### Projections collected at five angles were sufficient to capture the basic properties of RFs

A key consideration in calculating the FBP is the number of angles over which integral projections are collected: increasing the number will give a reconstruction of better quality but will also increase the time it takes to acquire the necessary data. We explored this trade-off using the same model RF shown in Fig.1*A*. Using two orthogonal angles of projections we were able to estimate the location of the RF, but its oval shape was not apparent (Fig.3*A*). With five projections spaced 36 deg apart, we could estimate the centre, size, shape and orientation of the original field, but some streaking artefacts from the back projection remained. Additional projections did not seem to significantly increase the amount of spatial information that we could extract, but they did reduce streaking.

We quantified the quality of the RF reconstructions in two ways. The first was by summing the squared differences between the reconstructed field and the original model (Fig.3*B*, black curve), where the quality of the reconstruction increased in a log–linear fashion with number of angles. The second approach was more practical, and concentrated on the parameters that we sought to extract from the RF: its centre, sensitivity, spatial dimensions and orientation. These parameters were estimated using a 2-D Gaussian fit to the reconstructed RF (Fig.3*A*, red contours). Figure3*B* plots the sum of the squared differences between the fit and the original model. The goodness of fit increased dramatically up to four equally spaced angles of projection and then plateaued after five (Fig.3*B*, grey curve). It appears that implementing the filtered back projection with five equally spaced angles should be sufficient to recover the basic features of RF centres.

### Comparison of spatial RFs estimated by FBP and STA

To test for any systematic biases of the FBP method compared to reverse-correlating spikes with the stimulus, we reconstructed 78 RGC RFs by both methods using an MEA. We used 33 min of binary white noise to ensure adequate recovery of RFs with STA, and averaged 10 presentations of the stimulus for FBP, which took 13.5 min. Three example cells are shown side-by-side in Fig.4*A*, where the images have the same scaling, and map to exactly the same visual area. The fields measured with either method appear similar in their centre, size and shape regardless of the field's location on the sampled region. The estimates of RF centres were compared by finding the image coordinates with the largest magnitude pixel value. Figure4*B* shows histograms of the difference in *x* and *y* coordinates between the two methods of RF reconstruction: there was no significant difference.

Next, the extent of the RF was quantified by fitting with 2-D Gaussian surfaces. This provided the major and minor axes at half-maximum amplitude, and plots of the difference in the axis half-width obtained by the two methods are shown in Fig.4*C*. The FBP method returned a slightly larger value for the major axis (median difference 21 μm, inter-quartile range (IQR) 7 to 58 μm) whereas there was no significant difference for the minor axis (median difference 4.5 μm, IQR –6 to 9). These results demonstrate that no systematic bias was present when the parameters of the spatial RF are estimated with FBP as compared to STA.

A key practical issue during an experiment is the time it takes to map RFs: the longer the period over which responses are measured, the better the quality of the reconstruction. To compare how FBP and STA performed as a function of time, we estimated the SNR by comparing the amplitude of the RF centre to a measurement of the mean and standard deviation made outside the RF centre (see Methods). Figure4*D* shows that the SNR estimated using FBP was 2–3 times that using STA for equivalent durations of stimulation up to ∼15 min. The SNR achieved with three presentations of the FBP stimulus over a period of 4 min achieved the same SNR (12.7 ± 1.3) as white noise applied for ∼23 min (Fig.4*D*). We conclude that FBP is a more time-efficient method of estimating visual RFs than the STA.

### Estimating the impulse response using the FBP protocol

In addition to having different spatial RFs, visual neurons also vary in their temporal properties, which can be characterized by the time course of their responses to an impulse (Meister *et al*. 1994; Chichilnisky, 2001). We therefore developed a simple approach to estimate the impulse response from an FBP data set. For each RGC, the PSTH was divided into discrete time bins of 8 ms to generate a series of Radon transforms at different time delays, which were then used to generate a stack of images corresponding to evolution of the RF at 8 ms time intervals. Examples of this calculation for two cells are shown in Fig.5*A*, with the spike time histogram for each bar position at one angle shown on the left, the reconstructed RF in the centre, and the temporal response for different locations over the RF to the right. Movie S1 in the online Supporting information shows how the response to a step in contrast evolved over both space and time for the lower cell in Fig.5*A*. The impulse response was then obtained by differentiating the response to a step, and these are shown by the black traces in Fig.5*B*.

The SNR of the impulse response calculated from flashed bars depended on the width of the time bins used for the reconstruction, with improvements in SNR coming at the cost of temporal resolution. An advantage of the FBP approach is that this trade-off could be decided *post hoc*, and in Fig.5 we used 8 ms time bins so that the impulse response was sampled at 125 Hz. Using a protocol lasting 4 min, this temporal resolution allowed observation of the delay between stimulus onset and the beginning of the response as well as the very rapid transition from the minimum to maximum change in spike rate. These features of the impulse response were not evident when calculated from the STA after 33 min of white noise stimulation because the refresh time of the hardware delivering the stimulus was 33.3 ms (grey traces in Fig.5*B*). The FBP protocol therefore provided two distinct advantages over STA in assessing the temporal characteristics of RFs: the impulse response was measured directly from the step response, and the time resolution achieved from a protocol lasting a few minutes provided basic information that was not apparent from the STA. Visual displays refreshing at 100–200 Hz are now available, so the STA could achieve similar temporal resolutions, although this is likely to come at the cost of much reduced SNR over similar periods of observation.

### Separation of ON and OFF components in a receptive field

Many cells in the visual system have receptive fields that contain both ON and OFF components, and these often overlap within their RF centres (Hubel & Wiesel, 1959; Martinez *et al*. 2005; Emran *et al*. 2007). A simple STA does not reveal the RF centre of such ON–OFF cells, as the two components will be averaged together. The FBP method, however, was able to separate the ON and OFF components of a single cell and reconstruct these RFs independently. An example of responses from a mixed ON–OFF ganglion cell in the retina of a goldfish is shown by the PSTH in Fig.6*A*: a burst of spikes occurred during a 100 ms presentation of a dark bar and then again after it was removed. These two response components were separated and used to construct distinct Radon transforms (Fig.6*B*). The RFs subsequently calculated by FBP were distinct, with a small central OFF component surrounded by a larger ON component (Fig.6*C*). The impulse response for the central ON component had a much longer latency than the OFF (Fig.6*F*): the OFF response, including the rebound, was complete by the time the ON response began. In comparison, the STA of the same cell did not reveal the ON component of the RF (Fig.6*E* and *F*). A second example of this analysis, also for an ON–OFF cell, is shown in Fig.2. In this case the ON and OFF components occurred with similar lags. These results demonstrate how the stimulus protocol used to estimate RFs by FBP allows easy separation of ON and OFF response components in both the spatial and temporal dimensions.

### FBP captures more ganglion cell types than STA

White noise is not an effective stimulus for all visual neurons and some respond much more strongly to structured stimuli (Marre *et al*. 2012; Talebi & Baker, 2012). We therefore compared the number of RF fields that could be mapped using the STA and by FBP. From the data featured in Fig.4, we were able to reconstruct 97 RFs using an FBP protocol of 13.5 min, but only 78 using 33 min of white noise stimulation followed by calculation of the STA. The 19 cells that were detected by FBP but not STA were of two basic types: 12 were orientation-selective and 7 were weakly responding. Orientation-selective RGCs were clearly visible in the Radon transform from which orientation tuning could be immediately calculated, as shown in Fig.7*A*. Stationary or moving bars orientated orthogonal to the preferred angle elicited much weaker responses (Fig.7*A* and *B*) and the RFs of these orientation-selective neurons were elliptical when reconstructed by FBP (Fig.7*C*). It appears that a flickering checkerboard stimulus did not activate orientation-selective neurons efficiently. The remaining 7 RGCs absent from the STA reconstructions were all characterised by small increases in firing rate upon stimulation, averaging only 7.7 ± 1.9 Hz (Fig.7*D*). It is possible that the STA method may have recovered these RFs if the acquisition time had been longer. These results demonstrate that the FBP provides a sensitive method for reconstructing receptive fields, detecting both weakly responding cells and describing the tuning of orientation-selective cells.

### FBP can map receptive fields by optical imaging of activity

Advances in multiphoton microscopy and fluorescent reporter proteins are now making it possible to image the activity of many neurons simultaneously *in vivo* (Marvin *et al*. 2013; Chen *et al*. 2013). This approach holds great promise for analysing the function of neural circuits at the population level, and is being applied particularly usefully to the retina (Odermatt *et al*. 2012; Esposti *et al*. 2013; Yonehara *et al*. 2013; Nikolaev *et al*. 2013; Park *et al*. 2014) and visual cortex (Katona *et al*. 2012; Scott *et al*. 2013). We therefore investigated how far the FBP approach could be used to map RFs by imaging calcium reporter proteins in the retina of zebrafish. Rather than image calcium in somata or dendrites, we used a synaptically targeted GCaMP6 to measure Ca^2+^ changes in the presynaptic terminals of bipolar cells (Dreosti *et al*. 2009; Chen *et al*. 2013).

A larval zebrafish (8 dpf) was positioned in front of a screen, as shown in Fig.8*A*, and bars covering 6.2 deg of visual angle were flashed with 50% overlap, giving a resolution of 3.2 deg of visual angle. A field of view containing the inner plexiform layer labelled with SyGCaMP6(10.500) was scanned at 10 Hz while each bar was flashed for 0.5 s with 2 s between each presentation. The response of a single bipolar terminal to the series of flashed bars is shown in Fig.8*B*, the complete series lasting 215 s. We then applied the same FBP protocol used to reconstruct RFs from electrophysiological measurements (Fig.2): responses were parsed and deshuffled into a PSTH for each angle (Fig.8*C*) and a Radon transform was generated from the PSTH using the integral of the responses (Fig.8*D*), allowing the RF of this bipolar cell terminal to be recovered with FBP (Fig.8*E*). The same procedure was applied to the 94 responding terminals within the field of view shown in Fig.8*F*, and the locations of their RF centres are shown in Fig.8*G* relative to the area of visual space that was probed. As would be expected, the position of the RF in visual space varied according to the terminals’ lateral location in the inner plexiform layer (Fig.8*F*). These results demonstrate that the FBP method is easily implemented to map the RFs of visual neurons in imaging experiments and that it should prove useful in other visual areas.

The results in Fig.8 highlight an important advantage of imaging compared to electrophysiology: the ability to assess activity within particular neuronal compartments while observing a population of neurons. Mapping the RFs of bipolar cell terminals is of particular interest because these compartments are the only route through which the visual signal reaches the inner retina. The computations performed by synapses are difficult to assess by recording from the soma, so it is important to monitor their activity directly (Abbott & Regehr, 2004; Baden *et al*. 2011; Schnell *et al*. 2014). Furthermore different synaptic terminals from a single bipolar cell might transmit diverse signals to downstream neurons (Asari & Meister, 2012, 2014). The FBP method combined with imaging therefore opens the way to investigating how the output of neurons in the visual system is shaped by the circuit in which they are embedded.

## Discussion

We have described a new method for the rapid mapping of visual RFs using flashed bars with filtered back projection and demonstrated its utility when measuring neural responses electrophysiologically and with fluorescent reporter proteins. This approach allows RFs to be mapped rapidly; averaging three responses at each of five angles recovers the RFs within 4 min. Crucially, we demonstrate that use of FBP also allows RFs to be reconstructed from population imaging data *in vivo*, and in neuronal compartments as small as synaptic terminals, again within 3–4 min. We suggest that the FBP method will be generally useful in both electrophysiological and imaging experiments monitoring neural activity at any stage of the visual system, from the retina to the cortex.

### Advantages of using FBP

The FBP method offers several advantages over use of the STA for recovering the spatio-temporal properties of a neuron's RF.

- **Speed of reconstruction**. Receptive fields can be mapped in 4 min and with an SNR matching 20 min of white noise (Fig.4*D*).
- **Time resolution**. The impulse response can be recovered with a temporal resolution of ∼125 Hz using an FBP protocol lasting a few minutes. This is an improvement over the STA, where the impulse response is limited by the refresh rate of the monitor, typically 30–60 Hz. As a result, FBP defined the latency and time course of the impulse response more precisely than the STA (Fig.5). The impulse responses obtained by STA also appear slower than those from the FBP method, possibly because the STA assumes that spikes arise from an inhomogeneous Poisson process – an assumption that can result in significant deviations of the estimated impulse response from its true value (Pillow & Simoncelli, 2003).
- **Separation of ON and OFF components**. Visual neurons often have overlapping ON and OFF components to their receptive fields (Hubel & Wiesel, 1959; Martinez *et al*. 2005). Using flashed bars combined with FBP, we measured the ON and OFF components of a spatial RF independently and derived their corresponding impulse responses simply by differentiation of the step response (Fig.6). ON and OFF components are harder to separate using the STA because these are averaged together, resulting in an ambiguous map and impulse response. More complex analyses are required to separate ON and OFF components, such as spike-triggered covariance (Cantrell, 2010; Zhang *et al*. 2012), although these have the advantage of probing stimulus space for more complex features that drive spiking (Fairhall, 2006). The use of FBP to separate ON and OFF components could be improved further by mapping the RF with both dark and bright bars to ensure that the effective contrast for each transition is equal. In this study we used dark bars because OFF responses predominate in RGCs of fish (Emran *et al*. 2007).
- **Complex RFs**. FBP recovered more ganglion cell RFs than the STA method, which is consistent with reports that some RGCs are not stimulated effectively by a white noise stimulus (Marre *et al*. 2012). Weak responses were not, however, the only reason for some RFs being ‘hidden’ to a white-noise stimulus: FBP also recovered the RFs of orientation-selective RGCs that were not detected by the STA (Fig.7). We are unsure why the STA should be particularly ineffective in detecting orientation-selective RGCs, especially given that STA detects orientation-selective neurons in V1 of mice as effectively as gratings (Niell & Stryker, 2008). One possibility is that the high contrast stimuli used in our study generates stronger lateral inhibition to orientation-selective RGCs.

### Application of FBP to optical measurements of neural activity

The FBP algorithm can be used to reconstruct RFs from measurements of neural activity made *in vivo* using multiphoton microscopy (Fig.8). Currently, the most useful reporters for optophysiology are the genetically encoded calcium indicators, such as the GCaMPs (Marvin *et al*. 2013; Chen *et al*. 2013), but these do not achieve single spike resolution, especially if spike rates are greater than a few hertz (Chen *et al*. 2013). This poses a problem for STA analysis, which has been addressed by different means. Smith & Häusser (2010) deconvolved fluorescent signals to estimate spike times using a kernel calculated from separate electrophysiological measurements of spike rates. A second approach, used by Bonin *et al*. (2011), is to measure the average stimulus preceding optical events of different amplitudes (representing bursts of spikes of variable number), and then weighting the event-triggered stimulus ensemble according to the amplitude of the fluorescence change.

We sought a time-efficient approach for the mapping of RFs across populations of neurons observed by multiphoton imaging *in vivo* and found that this could be achieved using flashed bars and the FBP algorithm in neuronal compartments as small as synaptic terminals (Fig.8). The FBP method circumvents the problem of slow optical reporters because it integrates the total recorded activity of the cell following each stimulus event. An alternative approach to mapping RFs using optophysiology may be to use a white-noise stimulus and reverse-correlate continuous signals from, for instance, a calcium reporter. This basic approach has been used to map RFs of non-spiking neurons from continuous measurements of membrane potential (Olveczky *et al*. 2003; Baccus *et al*. 2008). We suspect, however, that the duration of the white-noise stimulus required to reconstruct RFs will be longer than that required for the STA.

In summary, the use of flashed bars and the FBP algorithm can rapidly reconstruct the RF of neurons in the visual system, and is applicable to both electrophysiological and optical experiments. Measuring RFs of neuronal compartments such as synaptic terminals should advance our understanding of how complex computations are generated by biological circuits.

## Key points

- To understand vision, we must measure the spatio-temporal receptive field of neurons in the visual system.
- We describe how the filtered back projection can be used to map the receptive fields of many neurons simultaneously, within a few minutes.
- This method can also reveal complex features of visual receptive fields such as the tuning of orientation selective neurons and the contributions from separate ON and OFF components.
- We demonstrate that the filtered back projection is suited to mapping receptive fields from populations of neurons recorded with imaging or electrophysiology and should therefore prove useful for investigations of visual processing throughout the visual pathway.

## Abbreviations

FBP: filtered back projection
MEA: multi-electrode array
PSTH: peri-stimulus time histogram
RF: receptive field
RGC: retinal ganglion cell
SNR: signal-to-noise ratio
STA: spike-triggered average

## Supporting Information

The following supporting information is available in the online version of this article.

Movie S1 shows the spatio-temporal receptive field of the OFF cell in the lower panels of Fig. 5. The time of the stimulus is indicated by the square in the bottom left. The frame rate represents 1/20th of real time.
