# Supplementary material for: Rapid mapping of visual receptive fields by filtered back projection: application to multi-neuronal electrophysiology and imaging
Source: J Physiol. 2014 Sep 29;592(Pt 22):4839–54. doi: 10.1113/jphysiol.2014.276642 (PMC4259530; doi:10.1113/jphysiol.2014.276642)
Supplement: Supplementary file 1 [file tjp0592-4839-sd1.zip › tjp6334-sup-0001-text.rtf]

Supporting Movie1 Legend

The movie shows the spatio-temporal RF of the OFF cell in the lower panels of Fig. 5. The time of the stimulus is indicated by the square in the bottom left.  The field of view in the movie and the spatial RF shown in Fig. 5 are the same. The frame rate represents 1/20^th^ of real time.
